# AI-driven adaptive treatment strategies in internet-delivered CBT

**DOI:** 10.1192/j.eurpsy.2021.75

**Published:** 2021-08-13

**Authors:** V. Kaldo, N. Isacsson, E. Forsell, P. Bjurner, F. Ben Abdesslem, M. Boman

**Affiliations:** 1 Clinical Neuroscience, Center For Psychiatry Research, Karolinska Institutet, Stockholm, Sweden; 2 Eecs/scs/mcs, Royal Institute for Technology, Stockholm, Sweden

**Keywords:** Adaptive Treatment Strategy, machine learning, prediction, Internet CBT

## Abstract

**Introduction:** Adaptive Treatment Strategies warns therapists of patients at risk of treatment failure to prompt an adaption of the intervention. Internet-delivered Cognitive Behavioural Therapy (ICBT) collects a wide range of data before and during treatment and can quickly be adapted by adjusting the level of therapist support. **Objectives:** To evaluate how accurate machine learning algorithms can predict a single patient’s final outcome and evaluate the opportunities for using them within an Adaptive Treatment Strategy. **Methods:** Over 6000 patients at the Internet Psychiatry Clinic in Stockholm receiving ICBT for major depression, panic disorder or social anxiety disorder composed a training data set for eight different machine learning methods (e.g. k-Nearest Neighbour, random forest, and multilayer perceptrons). Symptom measures, messages between therapist and patient, homework reports, and other data from baseline to treatment week four was used to predict treatment success (either 50% reduction or under clinical cut-off) for each primary symptom outcome. **Results:** The Balanced Accuracy for predicting failure/success always were significantly better than chance, varied between 56% and 77% and outperformed the predictive precision in a previous Adaptive Treatment Strategy trial. Predictive power increased when data from treatment weeks was cumulatively added to baseline data. **Conclusions:** The machine learning algorithms outperformed a predictive algorithm previously used in a successful Adaptive Treatment Strategy, even though the latter also received input from a therapist. The next steps are to visualize what factors contributes most to a specific patient’s prediction and to enhance predictive power even further by so called Ensemble Learning.

**Disclosure:**

No significant relationships.

